# SCIBER: a simple method for removing batch effects from single-cell RNA-sequencing data

**DOI:** 10.1093/bioinformatics/btac819

**Published:** 2022-12-22

**Authors:** Dailin Gan, Jun Li

**Affiliations:** Department of Applied and Computational Mathematics and Statistics, University of Notre Dame, Notre Dame, IN 46556, USA; Department of Applied and Computational Mathematics and Statistics, University of Notre Dame, Notre Dame, IN 46556, USA

## Abstract

**Motivation:**

Integrative analysis of multiple single-cell RNA-sequencing datasets allows for more comprehensive characterizations of cell types, but systematic technical differences between datasets, known as ‘batch effects’, need to be removed before integration to avoid misleading interpretation of the data. Although many batch-effect-removal methods have been developed, there is still a large room for improvement: most existing methods only give dimension-reduced data instead of expression data of individual genes, are based on computationally demanding models and are black-box models and thus difficult to interpret or tune.

**Results:**

Here, we present a new batch-effect-removal method called SCIBER (Single-Cell Integrator and Batch Effect Remover) and study its performance on real datasets. SCIBER matches cell clusters across batches according to the overlap of their differentially expressed genes. As a simple algorithm that has better scalability to data with a large number of cells and is easy to tune, SCIBER shows comparable and sometimes better accuracy in removing batch effects on real datasets compared to the state-of-the-art methods, which are much more complicated. Moreover, SCIBER outputs expression data in the original space, that is, the expression of individual genes, which can be used directly for downstream analyses. Additionally, SCIBER is a reference-based method, which assigns one of the batches as the reference batch and keeps it untouched during the process, making it especially suitable for integrating user-generated datasets with standard reference data such as the Human Cell Atlas.

**Availability and implementation:**

SCIBER is publicly available as an R package on CRAN: https://cran.r-project.org/web/packages/SCIBER/. A vignette is included in the CRAN R package.

**Supplementary information:**

Supplementary data are available at *Bioinformatics* online.

## 1 Introduction

Single-cell RNA-sequencing (scRNA-seq) enables transcriptomic profiling at a single-cell resolution in a high-throughput manner (Fan *et al.*, 2020; Klein *et al.*, 2015; Macosko *et al.*, 2015; Rosenberg *et al.*, 2018; Zheng *et al.*, 2017). While scRNA-seq from a single experiment can be used to describe transcriptomic heterogeneity of different cell types, combining data from multiple datasets further enhances the power. However, there are often systematic differences between datasets called ‘batch effects’ that are caused purely by technical differences between datasets instead of biological variations. These technical differences include differences in scRNA-seq protocols, platforms, technologies (Hashimshony *et al.*, 2012; Klein *et al.*, 2015; Macosko *et al.*, 2015; Picelli *et al.*, 2014; Ramsköld *et al.*, 2012), read processing, quality control, normalization procedures (Stegle *et al.*, 2015), as well as different donors, capturing time and handling personnel (Fan *et al.*, 2020; Korsunsky *et al.*, 2019; Tran *et al.*, 2020). Batch effects are often substantial in scRNA-seq data; they may confound meaningful biological variations, lead to false discoveries and mislead the interpretation of the data (Hicks *et al.*, 2018; Leek *et al.*, 2010). Although ingenious experimental designs have been recommended to minimize batch effects, such as pooling cells from multiple batches into a single one for sequencing (Cao *et al.*, 2017; Kang *et al.*, 2018; McGinnis *et al.*, 2019; Rosenberg *et al.*, 2018), practical issues arise from logistical and temporal limitations and sample acquisition (Fan *et al.*, 2020). As a result, computational methods that remove batch effects are often essential for unified single-cell transcriptomics analysis (Fan *et al.*, 2020; Welch *et al.*, 2019).

Current batch-effect-removal methods can be roughly divided into five categories: anchor-based, graph-based, anchor-graph-based, deep learning-based and model-based (Fan *et al.*, 2020; Forcato *et al.*, 2021). Below, we give examples or representatives in each category and briefly describe their ideas.

Anchor-based methods first decide pairs of cells that share similar expression pattern across batches, which are called ‘anchors’, and then use these pairs to simultaneously integrate batches and remove batch effects. The method mutual nearest neighbors (MNN)-Correct (Haghverdi *et al.*, 2018) proposes to use MNN to identify anchors, and this idea was then applied and extended by SMNN (Yang *et al.*, 2021), BEER (Zhang *et al.*, 2019a), Seurat (Stuart *et al.*, 2019), fastMNN (Lun, 2019), Scanorama (Hie *et al.*, 2019) and scMerge (Lin *et al.*, 2019).

Graph-based methods construct weighted graphs between and within batches and then use community detection methods to detect shared cell populations across batches. Conos (Barkas *et al.*, 2019) performs pairwise comparisons of batches to establish initial graphs between cells across different batches, creates a joint graph by combining inter-batch edges with lower-weight intra-batch edges and then applies community detection methods such as Walktrap, Louvain and Leiden, to the joint graph to decide clusters of different cell types. BBKNN (Polański *et al.*, 2020) first constructs graphs for each cell by finding its k-nearest neighbors in each batch independently and then merges sets of cell neighbors based on the distance metrics used in UMAP (Uniform Manifold Approximation and Projection) (McInnes *et al.*, 2018). scPopCorn (Wang *et al.*, 2019) relies on a co-membership propensity graph used in Google’s PageRank algorithm (Andersen *et al.*, 2006) to compute intra-dataset edges, uses cosine similarity matrix to define inter-dataset edges and then uses k-partitioning to decide shared populations across batches.

Anchor-graph-based methods make use of both anchors and graph representation. As a representative, LIGER (Welch *et al.*, 2019) first uses integrative non-negative matrix factorization to determine a low-dimensional space where each cell can be characterized by two sets of factors called dataset-specific factors and shared factors, and then constructs a neighborhood graph for the shared factors, which is then used to identify joint clusters across batches that serve as anchors, by connecting cells with similar factor loading.

Deep learning-based methods use deep neural networks to learn patterns in datasets. MMD-ResNet (Shaham *et al.*, 2017) assumes that batches differ in distribution and develops a residual network to learn a map that calibrates the distribution of the source batch to match that of the target batch. By combining transfer learning with variational auto-encoders, scGen (Lotfollahi *et al.*, 2019) first learns a distribution from the reference batch and then uses the trained network to predict the query dataset. SAUCIE (Amodio *et al.*, 2019) uses a deep neural network with autoencoders to learn the cellular manifold and obtains the batch-effect corrected data by one of its hidden layers. scAlign (Johansen and Quon, 2019) uses encoder networks to learn mappings from gene expression spaces of individual conditions into a common alignment space. scVI (Lopez *et al.*, 2018) uses a hierarchical Bayesian model with conditional distributions specified by deep neural networks to embed cells into a low-dimensional space, where batch effects have been removed.

Model-based methods impose assumptions on the distribution of (count or transformed) data or the clusters of cell types. ComBat (Johnson *et al.*, 2007) relies on an empirical Bayes framework. It assumes that the standardized gene expression of a given sample in a given batch follows a normal distribution with pre-specified prior distributions. Limma (Smyth and Speed, 2003) uses a linear model to fit the input data, and the linear model contains a blocking term to capture batch effects. ZINB-WaVE (Risso *et al.*, 2018) models the original count data by a zero-inflated negative binomial distribution. Harmony (Korsunsky *et al.*, 2019) develops a novel soft K-means method to cluster cells, which implicitly assumes a linear mixture model for the cell clusters.

Although many methods have been developed and demonstrated their abilities to remove batch effects in real data effectively, there is still a large room for improvement. First, most existing methods, no matter which of the five categories they belong to, do not output expression data in the original space/dimension, i.e. expression of individual genes. Instead, they give dimension-reduced data, where each dimension is typically a complex combination of many or all genes. While dimension-reduced data is often good enough for visualization, many other essential tasks of single-cell data analysis, such as differential expression analysis and pseudotime analysis, require expression data of individual genes. Second, many existing methods are based on computationally demanding models. As examples, graph-based methods often have a large algorithmic complexity, mixture non-Gaussian (e.g. zero-inflated negative binomial) models often require iterations and could have convergence issues [and also, many parametric models could be unsatisfactory to model real data, see e.g. Svensson (2020), Silverman *et al.* (2020) and Zhao *et al.* (2022) about the current debate on whether zeros are truly inflated in scRNA-seq data], and deep learning models are not only computationally demanding but also require GPU resources. Third, many existing algorithms rely on black-box models and thus are hard to interpret or tune. These models are complex and have a number of parameters that need to be optimized on individual datasets (e.g. the number of nodes in each hidden layer of a neural network and the learning rate when training a neural network), which can be very difficult for the user to tune, especially if these parameters do not have clear biological or statistical meanings.

In this article, we present a new method for batch-effect removal called Single-Cell Integrator and Batch Effect Remover (SCIBER) (pronounced as ‘cyber’) for short. SCIBER is a simple method that outputs the batch-effect corrected expression data in the original space/dimension. These expression data of individual genes can be directly used for all follow-up analyses. SCIBER has four steps, each having a clear biological meaning. The algorithms used for the four steps are k-means clustering, *t*-test, Fisher’s exact test and linear regression, respectively, all of which are easily comprehensible. The simplicity of SCIBER also makes it computationally light and scalable to data with a large number of cells. SCIBER requires minimum tuning: it has few tuning parameters and the default values work decently on a large range of datasets. We have shown that SCIBER, as a much simpler algorithm, performs comparably on real datasets or arguably better in some real datasets compared to the state-of-the-art methods.

There is one more feature of SCIBER: it is a ‘reference-based’ method. That is, one of the batches is treated as the ‘reference’ and kept untouched, and what SCIBER does is to ‘project’ other batches to the reference batch. Most existing methods are not reference-based: they are often done by projecting all batches to a new shared space, thus all batches have been changed during batch-effect removal and no batch has the luxury of staying the same. Both reference-based and non-reference-based are commonly used strategies in the literature on batch-effect removal in general, and they each have their own characteristics and advantages. For single-cell expression data, reference-based methods can be a better option when there is a natural choice of the reference batch, or when the ‘standard’ data are available and one wants to ‘add’ new data to them. For example, the Human Cell Atlas project (Regev *et al.*, 2017), which aims to build comprehensive reference maps of all human cells, provides high-quality data that can serve as calibrated reference batches. SCIBER can add other data, with batch-effect removed, to them. On the other hand, if a non-reference-based method is used, these ‘standard’ data, as well as other data, will be modified, and thus the visualization, differential expression, cell clusters and other results on these standard data will also change, which is often undesired.

## 2 Materials and methods

### 2.1 Overview

SCIBER takes scRNA-seq data from *B *+* *1 batches, including a reference batch and *B* query batches (B≥1). When not given, the reference batch can be assigned as the batch generated by the most reliable sequencing technique or protocol among the *B *+* *1 batches, the batch with the largest number of cell types or cells, the batch with the largest number of reads, etc. The scRNA-seq data is assumed to be pre-processed, e.g. normalized by the sequencing depth and log-transformed to stabilize the variance.

Keeping the reference batch untouched, SCIBER projects each query batch to the space of the reference batch. The projected query batches are the batch-effect corrected data, or ‘corrected data’ for short, which can be directly compared with the untouched reference data for downstream analyses such as differential expression analysis, or directly concatenated with the untouched reference batch for downstream analyses such as cell type identification or lineage analysis.

The basic idea of SCIBER, different from all other batch-effect-removal algorithms, is to match cell clusters across batches by differentially expressed (DE) genes. In another word, SCIBER assumes that cell clusters corresponding to the same cell type should have an essentially identical set of DE genes, despite the existence of batch effects. This idea is implemented in the following four steps.

Assume that the expression of *p* genes is measured in *n_b_* cells in batch *b* (b=0,1,…,B, and *b *=* *0 refers to the reference batch). Let $x_{ij}^{\left(b\right)}$ be the expression of gene *i* in cell *j* from batch *b*, where i=1,…,p, and $j=1,\ldots ,n_{b}$.

### 2.2 Step 1: K-means clustering for each individual batch

In Step 1, a K-means clustering is performed on each batch. That is, the *n_b_* cells in batch *b* are clustered into *K* clusters using K-means clustering, based on their expression on the *p* genes. By default, $K=\sqrt{n_{0}}$, where *n*_0_ is the number of cells in the reference batch.

### 2.3 Step 2: Marker gene identification

In Step 2, for each of the *K* clusters in each batch, DE genes are identified by a two-sample *t*-test, with one group being cells in the cluster and the other group being the other cells (i.e. all the cells not in this cluster). The *h* (by default, *h *=* *75) genes that are up-regulated with the smallest *P*-values are claimed as the ‘marker genes’ for the cluster.

### 2.4 Step 3: Fisher’s exact test for cluster alignment

For each cluster in the query batch (‘query cluster’ for short), Step 3 finds the cluster in the reference batch (‘reference cluster’ for short) that has the largest number of shared marker genes with it. These two clusters are called a ‘matched pair’. A *P*-value is assigned to each matched pair using a Fisher’s exact test. To be more specific, for each matched pair, a two-by-two contingency table is formed, with rows being whether a gene is in or not in the marker gene set of the query cluster and columns being whether a gene is in or not in the marker gene set of the reference cluster, and a *P*-value is given by a Fisher’s exact test on this contingency table.

Note that not every matched pair is ‘good enough’ or ‘true’. Biologically, some cell types are only present in the query batch but not the reference, so they should not have a match in the reference batch. SCIBER takes this into account and only keeps a subset of matched pairs, called ‘putative matches’. SCIBER keeps the *ω* proportion of matched pairs with the smallest *P*-values. Here, *ω* is a user-specified pre-fixed parameter between 0 and 1, and it has a clear biological meaning: roughly speaking, it is the proportion of cells that are present in both the query and the reference batch. SCIBER sets ω=0.5 by default.

### 2.5 Step 4: Projection of the query batch to the reference batch

Step 4 projects gene expression from each query batch to the reference batch based on the putative matches. Here is how the projection is made for each query batch. First, we compute its centroid in the query cluster and its centroid in the reference cluster. Each centroid is a vector with length *p*, with element *i* being the average expression of gene *i* for cells in the cluster.

Then, for each cell $j\in 1,\ldots ,n_{b}$ in the query batch, no matter whether it is in a cluster that has a putatively matched cluster or in a cluster that does not, we decompose its expression $x_{j}^{\left(b\right)}$ (on all genes, and so it is a length *p* vector) by a linear combination of the centroids of clusters that each have a putatively matched cluster. Let $X_{c}^{\left(b\right)}$ be a matrix whose columns are the centroids of clusters that each have a putatively matched cluster, where subscript *c* stands for ‘centroids’, and let $X_{c}^{\left(0\right)}$ be the corresponding matrix of the reference batch. The decomposition can be written as ${\hat{x}}_{j}^{\left(b\right)}=X_{c}^{\left(b\right)}{\hat{\beta }}^{\left(b\right)}$ with ${\hat{\beta }}^{\left(b\right)}={\left[{\left(X_{c}^{\left(b\right)}\right)}^{T}X_{c}^{\left(b\right)}\right]}^{-1}{\left(X_{c}^{\left(b\right)}\right)}^{T}x_{j}^{\left(b\right)}$. Here, ${\hat{x}}_{j}^{\left(b\right)}$ is the fitted expression. After the decomposition, the projected data for cell *j* (i.e. batch-effect removed expression of cell *j*), denoted as ${\tilde{x}}_{j}^{\left(b\right)}$, is obtained by $X_{c}^{\left(0\right)}{\hat{\beta }}^{\left(b\right)}$.

The above description is for each cell. Actually, this decomposition and projection can be done simultaneously for all cells in a batch by using a single formula ${\tilde{X}}^{\left(b\right)}=X_{c}^{\left(0\right)}{\left[{\left(X_{c}^{\left(b\right)}\right)}^{T}X_{c}^{\left(b\right)}\right]}^{-1}{\left(X_{c}^{\left(b\right)}\right)}^{T}X^{\left(b\right)}$, where $X^{\left(b\right)}$and ${\tilde{X}}^{\left(b\right)}$(without subscript *c*) are the raw and projected data matrices of batch *b*.

SCIBER is publicly available as an R package SCIBER on CRAN.

### 2.6 Some considerations regarding the algorithm

Setting the number of clusters *K* for the clustering algorithm (e.g. K-means) is often difficult when clustering single-cell data. For one reason, the number of cell types present in the data is often unknown. Moreover, even if the number of cell types is known, using it as *K* may still give poor clustering results. This is because different cell types often have quite unbalanced numbers of cells and there may be outliers in the data, and clustering algorithms often do not handle unbalanced clusters and outliers perfectly. For example, K-means clustering is sensitive to outliers and tends to combine small clusters and divide large clusters (Hastie *et al.*, 2009; Tan *et al.*, 2016). Fortunately, this difficulty barely matters in the K-means clustering in step 1 of SCIBER. The aim of applying K-means clustering in SCIBER is to divide cells into many small groups; within each group, the expression of cells is highly similar. In fact, SCIBER wants the number of groups to be significantly larger than the number of cell types so that a large cell type can be divided into multiple clusters, resulting in multiple centroids in Step 4 for a ‘high-resolution’ decomposition, while outliers form clusters by themselves and can then be left out from the putative matches in Step 3. SCIBER sets $K=\sqrt{n_{0}}$ by default.

Before figuring out the matching of cells (Step 3), SCIBER does all analyses, including the clustering in Step 1 and the DE analysis in Step 2, strictly separately for each batch. Any clustering or DE analysis on pooled data from multiple batches can be misled by batch effects.

In Step 3, SCIBER only assigns a subset of matched pairs as putative matches. This takes care of the important fact that some cell types are not present in both the reference batch and the query batch.

In Step 4, we use a simple linear model to project the query batch to the reference batch. We do not use a more complicated nonlinear model, such as a deep learning-based model or an ensemble decision tree, because a complicated model is not only often hard to tune and time-consuming to train but also likely to suffer from over-correction (Argelaguet *et al.*, 2021). On the contrary, our linear model has a closed-form solution without any tuning parameter, a high computational efficiency and a low risk of over-correction. Moreover, its linear nature can be highly appreciated when projecting cell types in the query batch that are absent in the reference batch. For example, if such a cell type lies in the very middle of two other cell types that are present in both the query and the reference batches, it will be decomposed as a combination of half and half from the two cell types, and in the corrected data, it will still be in the very middle of the two cell types. This is ‘interpolation’ in the linear model. Similarly, outliers will also be projected into appropriate locations by ‘extrapolation’ in the linear model.

## 3 Results

### 3.1 SCIBER removes batch effects effectively while preserving separation between cell types

We apply SCIBER in all the eight real datasets used in the review paper by Tran *et al.* (2020), except for a dataset from the human cell atlas, of which the cell type information is unavailable. This review paper systematically compared 14 batch-effect-removal methods from all the five categories on these real datasets. Their results show that Harmony (Korsunsky *et al.*, 2019), Seurat (v3) (Stuart *et al.*, 2019) and LIGER (Welch *et al.*, 2019) are the best methods in terms of accuracy. A summary of these eight datasets is given in Table 1, and details are given in Supplementary Material. It is worth noting that Dataset 5, which we downloaded from the review paper and contains 83 323 cells, is a subsampled dataset (the subsampling was done by the review paper) from the original dataset sequenced by Rosenberg *et al.* (2018) and Saunders *et al.* (2018), which contains 833 206 cells.

**Table 1.** Description of the eight datasets on which the batch correction algorithms were tested

No.	Dataset	No. of batches	No. of cells	Technologies	References
1	Mouse cell atlas	2	6954	Microwell-Seq Smart-Seq2	Tabula Muris Consortium *et al.* (2018) and Han *et al.* (2018)
2	Human PBMCs	2	15 476	10× 3′ 10× 5′	Zheng *et al.* (2017)
3	Human DCs	2	576	Smart-Seq2	Villani *et al.* (2017)
4	Mouse retina	2	71 638	Drop-seq	Macosko *et al.* (2015) and Shekhar *et al.* (2016)
5	Mouse brain	2	83 323	Drop-seq SPLIT-seq	Rosenberg *et al.* (2018) and Saunders *et al.* (2018)
6	Mouse HSPCs	2	4649	MARS-seq Smart-Seq2	Nestorowa *et al.* (2016) and Paul *et al.* (2015)
7	Human pancreas	5	14 767	inDrop CEL-Seq2 Smart-Seq2 SMARTer SMARTer	Baron *et al.* (2016), Muraro *et al.* (2016), Segerstolpe *et al.* (2016), Wang *et al.* (2016) and Xin *et al.* (2016)
8	Cell line	3	9531	10×	Hie *et al.* (2019) and Zheng *et al.* (2017)

PBMCs, peripheral blood mononuclear cells; DCs, dendritic cells; HSPCs, hematopoietic stem and progenitor cells.

The raw scRNA-seq data are normalized to remove the difference in sequencing depth and log-transformed to stabilize the variance. Then high-variable genes are identified for each batch, and genes that are not identified as high-variable in any of the batches are discarded. All of these pre-processing steps are conducted using the R package *Seurat* (Stuart *et al.*, 2019).

When applying SCIBER, we use the default values of all the parameters: $K=\sqrt{n_{0}}$ in Step 1, *h *=* *75 in Step 2 and ω=0.5 in Step 3. That is, a common set of parameter values are used for all datasets, and no tuning of parameters are involved.

We compare the performance of SCIBER with the three state-of-the-art methods reported by the review paper (Tran *et al.*, 2020): Harmony, Seurat and LIGER. Details of how these algorithms are used are given in Supplementary Material.

We use cLISI proposed by Korsunsky *et al.* (2019) as the numeric measure of performance. cLISI measures local cell-type purity: the idea is, in clear data, the cells should cluster according to the cell types, and thus the cell types in a cell’s neighbor should be quite ‘pure’. In Supplementary Material, we give the mathematical definition of cLISI and why a few other measures are less appropriate. Smaller cLISI values (values closer to 1.0) indicate higher local cell-type purities and are thus preferred.

However, cLISI has its limitations in measuring performance (reasons given in Supplementary Material). We also plot the batch-effect corrected data into a 2D space using UMAP and check the plot visually. Such a 2D plot contains much more information than any single numeric summary statistic and gives a more comprehensive picture of the performance.

**Fig. 1.**
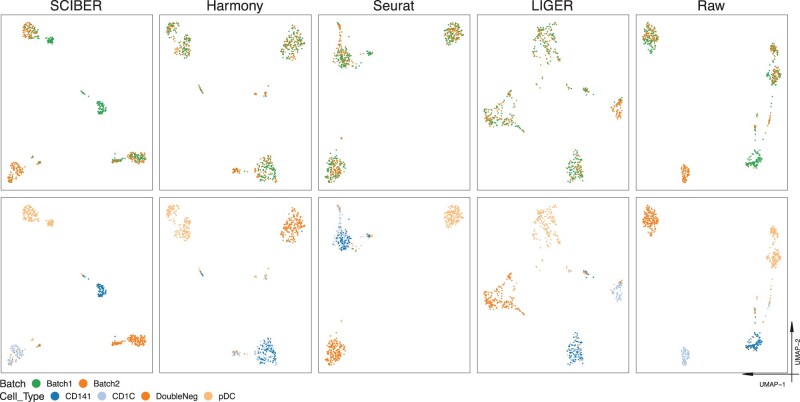
Scatter plots of cells from all batches of the human dendritic cell data (Dataset 3) on a 2D UMAP space. Different sub-plots show corrected data from different methods (from left to right: SCIBER, Harmony, Seurat and LIGER), as well as the raw (uncorrected) data. Cells in the first row of sub-plots are colored according to their batches, while cells in the second row are colored according to their actual cell types. It is clear that only SCIBER keeps CD141 and CD1C as two separated clusters, while the other three methods mix these two different cell types

Supplementary Tables S1–S8 give the cLISI scores for different methods in the eight datasets. The cell type cLISI score is the averaged cLISI score for a cell type, and the overall cLISI score is the average across the batch. We see that SCIBER performs comparably to the best performer of the other three methods in six out of the eight datasets, which is Seurat in some datasets and Harmony in the others, and significantly better than all the other three methods in the other two datasets (Datasets 3 and 6). Figure 1 and Supplementary Figures S1–S7 give the UMAP plots of all the datasets. The overall impression from the UMAP plots is consistent with that from cLISI.

Here, we use the human dendritic cells dataset (Dataset 3) as an example. The two batches of this dataset contain four different types of human dendritic cells (DCs): pDC (CD11C${}^{-}$CD123${}^{+}$ plasmacytoid DCs), DoubleNeg (CD11C${}^{+}$CD141${}^{-}$CD1C${}^{-}$ DC), CD141 (CD11C${}^{+}$CD141${}^{+}$ DC) and CD1C (CD11C${}^{+}$CD1C${}^{+}$ DC). Both Batches 1 and 2 contain 96 pDC cells and 96 DoubleNeg cells. Other than that, Batch 1 contains 96 CD141 cells, while Batch 2 contains 96 CD1C cells. Note that CD141 and CD1C are two cell types that are quite similar, so the main challenge in this dataset is to keep these two cell types separated while merging each of the other two cell types (pDC and DoubleNeg) from the two different batches. In this dataset, SCIBER achieves a much lower cLISI value (1.12) than those from the other methods (1.29 from Harmony, 1.43 from LIGER and 1.38 from Seurat). Figure 1 gives the 2D UMAP plots of the batch-effect corrected data from different methods, as well as the raw (i.e. uncorrected) data. Cells in the first row of sub-plots are colored according to their batches, and cells in the second row of sub-plots are colored according to the cell types they belong to. Ideally, the cells should be separated according to the cell type but not the batch. In the figure, this means that points of different colors in a plot in the second row (which denote different cell types) should form separate clusters, and at the same time, points of the same color in a plot in the second row (which denote the same cell type) should not form sub-clusters that each has a different color in the corresponding plot in the first row (which denote different batches). Following this guidance, we find that while all methods successfully keep pDC and DoubleNeg separated from other cell types, only SCIBER keeps CD141 and CD1C as two well-separated clusters. Meanwhile, Harmony, Seurat and LIGER mistakenly mix CD141 and CD1C, which indicates that they likely have incorrectly aligned cells from CD141 and CD1C. The success of SCIBER in separating CD141 and CD1C could be attributed to its disinterest in aligning all cell clusters across batches (Step 3 of the algorithm). Additionally, in LIGER, the CD1C cells are further divided into two sub-clusters, determined by which batch they come from, and one of the two sub-clusters is thoroughly mixed with the CD141 cells. Interestingly, the UMAP plots of the raw data indicate that batch effects in this data might be minor; it seems that methods other than SCIBER likely have misidentified a proportion of the differences between cell types as batch effects and removed them.

A detailed discussion of results on the other datasets is available in Supplementary Material. Overall, as we have summarized, SCIBER shows similar and sometimes better, accuracy in removing batch effects. What’s more, SCIBER has clear advantages in its computational load, etc., which are discussed in the following sections.

### 3.2 Computational efficiency of SCIBER

**Fig. 2.**
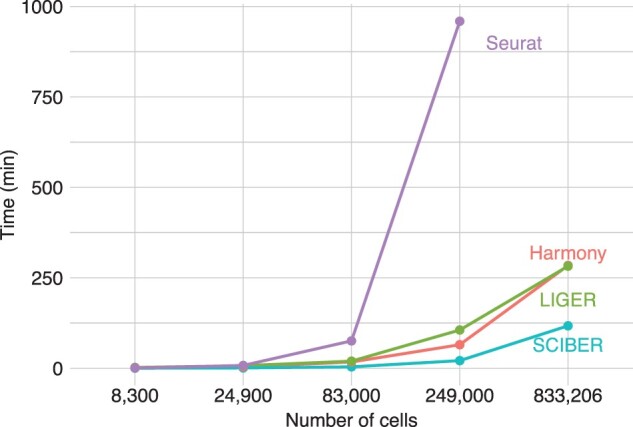
Runtime of different methods on the full and the down-sampled mouse brain data (Dataset 5). The full dataset contains 833 206 cells, and it is down-sampled to smaller numbers of cells. The runtime of Seurat on the full dataset is missing as it was terminated for excessive memory requests. It is clear that SCIBER has a significantly shorter runtime than all the other three methods

As a simple algorithm, SCIBER is computationally more efficient than Harmony, LIGER and Seurat, and it scales to datasets with a large number of cells. To show this, we choose the original full data of the mouse brain data (Dataset 5). We down-sample the number of cells from 833 206 to 249 000, to 83 000, to 24 900, and finally, to 8300, and record the runtime of SCIBER on an eight-core machine with 2.50 GHz CPU and 16 GB RAM. The results are shown in Figure 2. (There is no result for Seurat on the full dataset due to memory explosion.) We find that the computational time of SCIBER increases roughly linearly with the increase in the number of cells, although this could be a little hard to see from the figure, whose x-axis is on the log scale but y-axis is not. In general, SCIBER is 2.5 times faster than Harmony and LIGER, which in turn are much faster than Seurat.

### 3.3 SCIBER keeps the reference batch untouched while projecting query batches

As a reference-based method, SCIBER does not change the reference batch when removing batch effects from the query batches. This facilitates adding additional batches: when the additional batches are added, all downstream analyses on the reference batch, such as visualization (dimension reduction), differential expression analysis, regulatory network construction and pseudotime analysis, will remain unchanged.

**Fig. 3.**
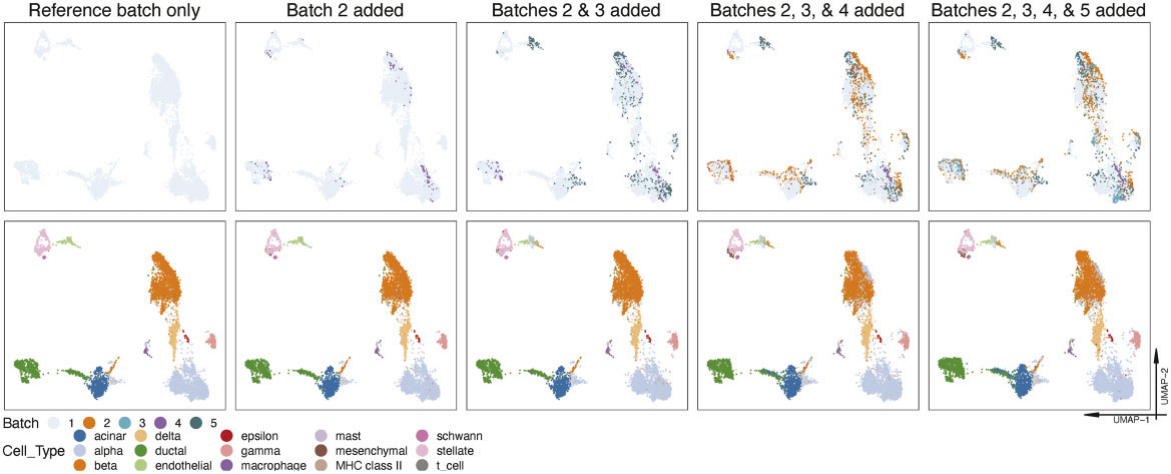
Multiple batch projection using UMAP visualization for the human pancreas data (Dataset 7). Cells in the first row of sub-plots are colored according to batch labels, while cells in the second row are colored according to cell type labels. The leftmost plots show the reference batch only, and the other plots from left to right show the batch-effect removed data when more batches are integrated by SCIBER. It is clear that the reference batch does not change when new batches are integrated

Using visualization as an example, Figure 3 demonstrates this point on the human pancreas data (Dataset 7), which contains human pancreas cells from five batches. We use the batch with the largest number of cells as the reference batch and integrate it with different numbers of other batches. As the plot shows, as new query batches are added, the reference batch will not change, as well as the query batches that already have been integrated.

### 3.4 SCIBER facilitates downstream analyses

The batch-effect corrected data obtained by SCIBER are the expression of individual genes, which can be directly used for downstream analyses. Here, we use pseudotime analysis and marker gene analysis on the human hematopoietic stem and progenitor cell data (Dataset 6) as an example. This dataset contains mouse hematopoietic stem and progenitor cells from two batches. Each batch contains common myeloid progenitor (CMP) cells, megakaryocyte–erythrocyte progenitor (MEP) cells, granulocyte–monocyte progenitor (GMP) cells and a few other types of cells. Biologically, these three cell types are of particular interest: CMPs may differentiate into either GMPs or MEPs, which form the cells of the granulocyte/macrophage or megakaryocyte/erythroid lineages, respectively (Akashi *et al.*, 2000). In the expression data, the clustering of cells is expected to reflect this developmental lineage, with GMPs and MEPs forming their respective clusters that are both close to CMPs (Haghverdi *et al.*, 2018). However, in the raw data, the cells are clustered mainly according to the batches they come from instead of their cell types (see the last column of sub-plots in Supplementary Fig. S5). This incorrect clustering is largely corrected by removing batch effects using any of the four methods, as shown in Supplementary Figure S5, indicating the success of these four methods. However, SCIBER is the only method among them that outputs the expression of individual genes.

**Fig. 4.**
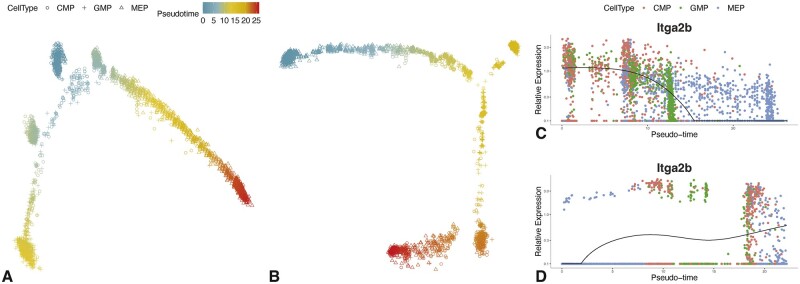
Pseudotime and marker gene expression on the mouse hematopoietic stem and progenitor cell data (Dataset 6). (**A**) Inferred pseudotime from output data of SCIBER. Each point represents a cell. The point types represent cell types, and the colors represent the inferred pseudotime. Starting from the top blue colored cells, there are two clear branches. (**B**) Inferred pseudotime from the raw data. The triangle points are broken into two far apart clusters, one in blue color and the other in red color. (**C, D**) The expression of gene Itga2b based on (C) the output data of SCIBER and (D) the raw data. According to the annotation database, this gene is a marker gene for both cell types CMP and GMP, but not a marker gene for MEP. The points are colored according to their actual cell types and are arranged from left to right according to the inferred pseudotime. The black curves are the fitted expression level along the pseudotime. In (C), most CMPs appear at the very beginning, GMPs appear in the middle, and MEPs appear at last. Also, the black curve shows a sudden drop, indicating a much lower expression level in MEPs compared to CMPs and GMPs. In (D), such an ordered arrangement of different cell types and a drop in expression are not observed

We pool the batch-effect corrected data by SCIBER from all batches and conduct a pseudotime analysis with R package *monocle* (Trapnell *et al.*, 2014). The result is shown in Figure 4 A, where each point corresponds to a cell, with its shape (circle, cross or diamond) representing the cell type and color representing the inferred pseudotime. It is clear that the cells form two branches, both starting at the CMP (the blue circles in the middle), one ending at GMP (yellow crosses on the left) and the other ending at MEP (red diamonds on the right). This correctly reflects the lineage. On the contrary, results from the same analysis in the raw data (Fig. 4B) contradict the biological fact. For example, the MEPs (diamonds), which are supposed to have large pseudotime values, form two groups, one (blue diamonds, on the top left) having very small pseudotime and the other having very large pseudotime (red diamonds, on the bottom left).

We further investigate how the expression of marker genes of these three cell types changes as a function of pseudotime. We use marker genes defined in the *CellMarker* database (Zhang *et al.*, 2019b). Figure 4C shows the expression of gene Itga2b on the batch-effect corrected data along the inferred pseudotime (x-axis). In the plot, each point represents a cell, with different colors presenting different cell types, and the black curve is the fitted (averaged and smoothed) expression level. We notice that most red points (CMPs) appear at the very beginning, green points (GMPs) appear in the middle, and blue points (MEPs) appear at last. This is consistent with the two branches observed in Figure 4A. Moreover, the black curve has a drastic drop as MEPs appear, indicating a much lower expression in MEPs. This reflects the biological truth: this gene is a marker gene (actually the only marker gene of this type in the *CellMarker* database) that is common for CMPs and GMPs but not for MEPs. Figure 4D shows the expression of Itga2b on the raw data, where its drop in MEPs has been distorted by batch effects. Supplementary Figures S8–S11 give plots for marker genes of other types, in which we have similar observations.

## 4 Discussion

We have presented SCIBER, a simple algorithm that removes batch effects from scRNA-seq data in a computationally efficient manner. While its accuracy is similar to, and sometimes better than, the state-of-the-arts, its ability to output expression data at the gene level facilitates follow-up analyses, and its reference-based nature makes it especially suitable for mapping data to standard reference datasets.

Unlike existing algorithms, SCIBER identifies matched cell clusters across batches according to DE genes. The four steps of the SCIBER algorithm rely on simple algorithms and/or tests, which do not impose strong assumptions on the distribution of the data or the shape of each cluster. (Although K-means clustering implicitly assumes each cluster has a round shape with similar sizes, SCIBER uses a large *K*, which breaks up large clusters and hence accommodates virtually any shape for the cell types.) The simplicity of the algorithm also makes it computationally fast. In our current implementation of SCIBER, the most time-consuming step is K-means clustering, and thus SCIBER can be further accelerated by replacing K-means with a more efficient clustering algorithm or using a more efficient implementation of K-means.

The SCIBER algorithm has three parameters: the number of clusters *K* in Step 1, the number of marker genes *h* in Step 2 and the proportion of matched clusters *ω* in Step 3. In Section 3, we have shown that no tuning of these parameters is needed for all the datasets we consider and the default values work well. To confirm the insensitivity of our method to the tuning parameters, we systematically study the effect of these tuning parameters. The results of different values of *K* ($0.8\sqrt{n_{0}},\;\sqrt{n_{0}}$ and $1.2\sqrt{n_{0}}$), *h* (50, 75 and 100) and *ω* (0.4, 0.5, 0.6 and 0.7) in all the eight datasets we consider are shown in Supplementary Tables S9–S11. It is clear that the performance of SCIBER changes little under different values of the parameters.

## Supplementary Material

btac819_Supplementary_Data
