# Supplementary material for: SCIBER: a simple method for removing batch effects from single-cell RNA-sequencing data
Source: Bioinformatics. 2022 Dec 22;39(1):btac819. doi: 10.1093/bioinformatics/btac819 (PMC9848058; doi:10.1093/bioinformatics/btac819)
Supplement: btac819_Supplementary_Data [file btac819_supplementary_data.pdf]

Supplementary Material for SCIBER: a simple method for removing batch effects from single-cell RNA-sequencing data

Dailin Gan and Jun Li*

Department of Applied and Computational Mathematics and Statistics, University of Notre Dame, Notre Dame, IN 46556, USA

* *To whom correspondence should be addressed. Tel: +1 574 631 3429; Fax: +1 574 631 4822; Email: jun.li@nd.edu*

1 A mathematical description about cLISI

The following description is summarized from the R and C++ code that implement cLISI, which are available at `https://github.com/immunogenomics/LISI`.

The computation of cLISI consists two steps. The first step is to use a Gaussian kernel to compute the probabilities of different cell types in a neighborhood. The size of the neighborhood is determined by a hyper-parameter U, the desired perplexity. The second step is to calculate the inverse Simpson's Index. Details are given below.

Given the number of cells N and the desired perplexity U ($U = 30$ by default), first one computes the distance matrix $\mathbf{D}$ of cells to their neighbors, which has K rows and N columns. Each entry in $\mathbf{D}$ is the distance from a cell to one of its nearest neighbors. K is the number of neighbors to be considered. In cLISI, K is set as $K = 3U$. The probability of cell type c in the neighborhood of cell j is defined as

$$p_j(c) = \sum_{i=1}^{\mathcal{I}_c} \frac{\exp(-\beta \mathbf{D}_{i,j})}{\sum_{k=1}^{K} \exp(-\beta \mathbf{D}_{k,j})}, \tag{1}$$

where $\mathcal{I}_c$ is the set of cells in cell type c in the neighborhood, and β is a parameter selected

by a binary search algorithm so that the following condition about perplexity is satisfied

$$\left|\log\left(\sum_{k=1}^{K}\exp(-\beta\mathbf{D}_{k,j})\right)+\beta\frac{\sum_{k=1}^{K}(\mathbf{D}_{k,j}\bmod\exp(-\beta\mathbf{D}_{k,j}))}{\sum_{k=1}^{K}\exp(-\beta\mathbf{D}_{k,j})}-\log(U)\right|\leq 10^{-5}.$$

After getting the probability $p_j(c)$, the cLISI score for cell j is the inverse Simpson's index,

$$\text{cLISI}_j = 1/\sum_{c=1}^{C} p_j(c),$$

where C is the number of cell types in the K nearest neighbors of cell j.

2 Why using cLISI as the numeric measure of performance

In an ideal batch-effect-removed data, the cells should be clustered/separated by the cell types they belong to, not the batches they come from. Thus, if we pick up a randomly chosen cell and its neighbors (i.e., looking "locally"), we would expect that these cells are from the same cell type but different batches. In another word, a clean data should have high local cell-type purity and high local batch diversity. Thus, measuring the local cell-type purity and the local batch diversity have been two commonly used ways to assess batch-effect-removal methods in the literature.

However, we argue that local batch diversity is not a good measure when different batches have different compositions of cell types. To show this point, we consider data where cell type A only appears in batch 1 but not batch 2. Suppose we have a perfect algorithm that removes the batch effect completely while keeping different cell types separated. Then in the resulting data, neighbors of cells from cell type A should also be cells from cell type A and thus all come from batch 1. Hence, these neighbors have zero batch diversity. As a result, this perfect batch-effect-removal algorithm will not be favored when using high local

batch diversity as the criterion of performance. Instead, such criterion prefers algorithms that mistakenly mix cell type A with cells from other cell types so that the two batches are also mixed.

Using local cell-type purity to assess performance, in contrast, does not have such strong assumptions on the cell-type compositions. The measures proposed for cell-type purity include cLISI, ASW (average silhouette width) [14], and ARI (adjusted rand index) [6]. However, both ASW and ARI have serious limitations. ASW computes the silhouette score of a cell by comparing the average distance between this cell and the other cells in the same cluster and the average distance between this cell and all other cells in each of the other clusters. Unlike cLISI, which also relies on the choice of the distance metric but only uses distances for small neighborhoods, as given in Equation 1, ASW uses distances between faraway points (actually, the long distances determine the value of ASW), the value of ASW relies heavily on the choice of the distance metric. scRNA-seq data are high-dimensional, and using ASW with different distance metrics can lead to different conclusions on the best algorithm. ARI, on the other hand, computes the percentage of matches between the vector of true cell-type labels and the vector of cell-type labels inferred by a clustering algorithm of the user's choice. Since different clustering algorithms typically give quite different clustering results on scRNA-seq data, the conclusion made based on ARI depends heavily on the choice of the clustering algorithm. In contrast, cLISI does not rely on a distance metric or a clustering algorithm, making it a more objective measure of performance.

However, it is worth mentioning that having a good cLISI score, or any other numeric metric of local cell-type purity, is insufficient for claiming that a dataset is free from batch effects. Particularly, in an extreme case, if cell types are well separated in each batch, but different batches are also entirely separated, then such a dataset still has a perfect cLISI score. Thus, although we are using the cLISI as our numeric metric of performance, we should still check the UMAP plot, which contains more information about the data and gives a more comprehensive evaluation of the performance.

3 Datasets

1. Mouse cell atlas. This dataset contains two batches of mouse cell atlas. One was generated by Han et al. [4] with Microwell-Seq, and the other was generated by Tabula Muris Consortium [2] with 10x Genomics and Smart-Seq2 protocols. We use the batches cleaned by [19]. Both of them contain 15,006 genes and 11 cell types from diverse organ systems. There are 4,239 and 2,715 cells for each batch. The purpose of this dataset is to measure the performance of batch correction methods when different scRNA-seq technologies are used.

2. Human peripheral blood mononuclear cell. This dataset has two batches consisting of human PBMC scRNA-seq data [25]. The two batches are generated by the 3' and 5' 10X Genomics protocols which capture different regions of mRNA. Cell type annotation follows the annotation in Polanski et al. [12]. Only cells that are annotated by Polanski et al. are kept. The finalized dataset has 8098 cells for the 3' batch and 7378 cells for the 5' batch. Each batch has 17,430 genes. This dataset is to assess the integration ability of batch-effect correction methods when protocol-driven biological differences are provided.

3. Human dendritic cells. This dataset is composed of two batches of human blood dendritic cell (DCs) scRNA-seq data from [21]. Both batches have 288 cells and 16,594 genes with non-identical cell types. Both of them have 96 pDC and 96 double negative cells, while one has 96 CD141 cells and the other has 96 CD1C cells. This dataset is aimed to challenge batch correction methods to integrate common cell types across batches while keeping highly similar cell types separate.

4. Mouse Retina. This dataset contains two batches of mouse retina data generated by the Drop-seq technology from two unrelated laboratories [8, 17]. The batch from Shekhar et al. [17] has 26,830 cells, and the batch from Macosko et al. [8] has 44,808 cells. Both batches

have 12,333 genes. This dataset is to evaluate the performance of batch-effect correction methods when encountering a large dataset with non-identical cell types.

5. Mouse brain. This dataset contains two batches of mouse brain cells from Saunders et al. [15] and Rosenberg et al. [13], generated by two different technologies, the Drop-seq and SPLiT-seq protocols. The adjustment of cell type annotation and down-sampling procedures follow [19]. The first batch has 14,202 cells and the second one has 69,121 cells. Both batches have 17,745 genes. This dataset is to evaluate the performance of batch-effect correction methods on large batches obtained by different technologies.

6. Mouse haematopoietic stem and progenitor cells. This dataset has two batches, one from Nestorowa et al. [10] using the SMART-seq2 protocol , and the other from Paul et al. [11] using the MARS-seq protocol. The pre-processing and filtering workflow of both batches follows the procedure in Haghverdi et al.[3]. The choice of used cells and genes, and the modification of cell type labels follow the procedures in [19]. The finalized dataset has one batch with 1,920 cells, and another one with 2,729 cells. Both batches contain 3,467 genes. This dataset is to test the performance of batch correction methods on batches that are generated by different sequencing technologies and have non-identical cell types.

7. Human pancreas. This dataset contains five batches of human pancreatic data from five different sources [1, 9, 16, 22, 24]. Only genes that are present in all five batches are kept. The adjustment of cell type annotation follows [19]. The finalized dataset is composed of five batches generated by four different scRNA-seq technologies. Five batches have 14,767 cells, 15 cell types, and 15,558 genes in total. This dataset is to assess the performance of batch-effect correction methods across multiple batches.

8. Cell line. This dataset is composed of three batches from the cellranger output files of the cell line experiment [5, 25]. The first batch contains 2,885 293T cells, the second batch

contains 3,258 Jurkat cells, and the third batch contains a 50/50 mixture of Jurkat and 293T cells with 3388 cells. Each batch has 16,602 genes. This dataset is to measure the integration ability of each method across multiple batches.

4 Details of how we use other methods

For Harmony, we follow the suggested integration pipeline from the primary github repository provided by Harmony [7]. Pre-processing procedures for all batches are the same as those in SCIBER, and we use the top 20 principal components of each batch as the input of Harmony algorithm. We use the *HarmonyMatrix* function with $\theta = 1$ for all dataset analyses. All other parameters are kept as default.

For Seurat, we follow the suggested pipelines in the *Seurat* R package [18]. We use pre-processing steps as performed for SCIBER. We use functions $FindIntegrationAnchors$, $IntegrateData$, $ScaleData$ and $RunPCA$ to obtain Seurat embeddings with dimensions of 20. All the other parameters are kept as default.

For LIGER, we download the R package *rliger* and follow the integration pipelines for multiple scRNA-seq datasets [23]. We use pre-processing steps as performed for SCIBER. In the matrix factorization step involving the function *optimizeALS*, we choose $k = 20$, which specifies the inner dimension of factorization. We use the output of function *quantile_norm* as liger embeddings for further analysis. All the other parameters are kept as default.

For pseudotime analysis, we used the suggested pipelines in the *monocle* R package [20]. Since the projected query batches are assumed to be log-normalized, in the function *newCellDataSet*, we set $expressionFamily = uninormal()$ to accommodate this normalization. Since we use $uninormal()$, there is no need to use functions $estimateSizeFactors$ and $estimateDispersions$. In the dimensional reduction part, we set $norm\_method$ = "*none*", $pseudo\_expr = 0$ in the *reduceDimension* function. All the other parameters are kept as default.

5 Supplementary tables

Cell type cLISI	SCIBER	Harmony	LIGER	Seurat
Smooth-muscle/163 cells	1.2482	**1.1805**	1.2630	1.1941
Endothelial/1134 cells	1.0746	**1.0685**	1.1054	1.0704
Macrophage/469 cells	**1.2565**	1.3122	1.6052	1.3327
B-cell/1106 cells	1.0863	**1.0750**	1.3576	1.0758
Stromal/1032 cells	1.0541	1.0339	1.1019	**1.0333**
Dendritic/220 cells	**1.8038**	1.8563	2.0500	2.0783
NK/85 cells	1.6481	1.6907	1.7012	**1.5701**
Epithelial/390 cells	1.1188	1.1212	1.2618	**1.0730**
T-cell/1421 cells	1.1412	1.1332	1.1782	**1.0925**
Monocyte/480 cells	1.2008	1.1809	1.5399	**1.1622**
Neutrophil/454 cells	1.1302	1.1113	1.1710	**1.0982**
Overall cLISI	1.1483	1.1427	1.2775	**1.1371**

Table S1: Cell type and overall cLISI scores of the mouse cell atlas dataset (dataset 1) after removing batch effects using SCIBER, Harmony, LIGER and Seurat. The first column contains the cell types with the corresponding number of cells. A bold number stands for the best score in that row.

Cell type cLISI	SCIBER	Harmony	LIGER	Seurat
B cell/2371 cells	1.0124	1.0101	1.0167	**1.0080**
CD 4 T cell/4450 cells	1.2264	1.1046	1.0805	**1.0505**
Monocyte_CD14/4090 cells	1.1502	1.0262	1.0273	**1.0218**
CD 8 T cell/3142 cells	1.2589	1.1646	1.1193	**1.1045**
Monocyte_FCGR3A/561 cells	1.1502	1.1324	**1.1033**	1.1191
NK cell/593 cells	**1.0493**	1.0542	1.1379	1.0753
Megakaryocyte/106 cells	1.4979	1.5001	1.3346	**1.2010**
Plasmacytoid dendritic cell/139 cells	1.0474	1.0316	**1.0248**	1.0254
Hematopoietic stem cell/21 cell	1.7843	**1.3962**	1.8275	1.4949
Overall cLISI	1.1398	1.0832	1.0700	**1.0522**

Table S2: Cell type and overall cLISI scores of the human peripheral blood mononuclear cell dataset (dataset 2) after removing batch effects using SCIBER, Harmony, LIGER and Seurat. The first column contains the cell types with the corresponding number of cells. A bold number stands for the best score in that row.

Cell type cLISI	SCIBER	Harmony	LIGER	Seurat
DoubleNeg/192 cells	**1.0698**	1.0959	1.1225	1.0948
CD1C/96 cells	**1.1267**	1.7644	1.5822	1.7482
CD141/96 cells	**1.1725**	1.5097	2.1223	2.0339
pDC/192 cells	**1.1416**	1.1519	1.3055	1.1613
Overall cLISI	**1.1203**	1.2949	1.4268	1.3824

Table S3: Cell type and overall cLISI scores of the human dendritic cell dataset (dataset 3) after removing batch effects using SCIBER, Harmony, LIGER and Seurat. The first column contains the cell types with the corresponding number of cells. A bold number stands for the best score in that row.

Cell type cLISI	SCIBER	Harmony	LIGER	Seurat
bipolar/29779 cells	1.0288	1.0408	1.0598	**1.0123**
amacrine/4678 cells	1.1524	1.3007	1.1161	**1.0999**
muller/4569 cells	1.0732	**1.0201**	1.2051	1.0217
rods/29491 cells	1.0327	1.0338	1.0563	**1.0237**
cones/1916 cells	1.1036	1.0839	1.1209	**1.0562**
vascular/252 cells	1.2643	1.2836	1.6977	**1.1185**
ganglion/432 cells	1.4063	1.5024	1.6671	**1.1003**
horizontal/252 cells	1.2959	1.3752	1.8691	**1.0246**
pericytes/63 cells	2.0722	2.2595	2.4646	**1.5187**
astrocytes/54 cells	2.0509	2.1707	1.7391	**1.6971**
microglia/67 cells	1.4898	1.4579	1.7760	**1.2884**
fibroblasts/85 cells	1.7281	1.6789	2.2681	**1.1141**
Overall cLISI	1.0503	1.0626	1.0855	**1.0268**

Table S4: Cell type and overall cLISI scores of the mouse retina dataset (dataset 4) after removing batch effects using SCIBER, Harmony, LIGER and Seurat. The first column contains the cell types with the corresponding number of cells. A bold number stands for the best score in that row.

Cell type cLISI	SCIBER	Harmony	LIGER	Seurat
Neuron/56067 cells	1.0415	1.0251	1.0347	**1.0207**
Endothelial/4653 cells	**1.0534**	1.0736	1.1766	1.0708
Oligodendrocyte/11472 cells	1.0549	**1.0521**	1.0627	1.0552
Astrocyte/6736 cells	1.0982	1.0950	1.1203	**1.0569**
Mural/1281 cells	1.0756	1.1071	1.2193	**1.0689**
Microglia/572 cells	1.4879	1.5163	1.6339	**1.4470**
Polydendrocyte/1728 cells	1.2853	1.3819	1.3971	**1.2455**
Macrophage/174 cells	**1.6431**	1.7549	1.9010	1.6585
Ependymal/250 cells	1.1524	1.4344	1.4906	**1.1329**
Vascular/109 cells	1.9242	**1.7659**	1.8257	1.9316
Olfactory/18 cells	1.8968	2.4423	**1.7664**	1.8652
Choroid/9 cells	2.6261	2.6179	**2.3166**	2.4782
Mitotic/2 cells	**1.0834**	1.8668	2.8879	1.1782
Neurogenesis/252 cells	**1.6192**	1.7308	2.3194	1.6752
Overall cLISI	1.0621	1.0556	1.0763	**1.0447**

Table S5: Cell type and overall cLISI scores of the mouse brain dataset (dataset 5) after removing batch effects using SCIBER, Harmony, LIGER and Seurat. The first column contains the cell types with the corresponding number of cells. A bold number stands for the best score in that row.

Cell type cLISI	SCIBER	Harmony	LIGER	Seurat
GMP/1277 cells	**1.1743**	1.3747	1.4005	1.3753
MEP/1457 cells	**1.1907**	1.2317	1.2951	1.1974
CMP/808 cells	**1.7473**	1.9604	2.0533	2.0465
Unsorted/136 cells	**1.7402**	1.8410	1.8948	1.9165
LMPP/280 cells	**2.2369**	2.2900	2.5050	2.7857
MPP/368 cells	**2.2064**	2.2641	2.4879	2.7240
LTHSC/323 cells	1.8772	**1.8594**	2.1255	2.2090
Overall cLISI	**1.4901**	1.6045	1.6984	1.7017

Table S6: Cell type and overall cLISI scores of the mouse haematopoietic stem and progenitor cell dataset (dataset 6) after removing batch effects using SCIBER, Harmony, LIGER and Seurat. The first column contains the cell types with the corresponding number of cells. A bold number stands for the best score in that row.

Cell type cLISI	SCIBER	Harmony	LIGER	Seurat
alpha/5100 cells	1.0426	**1.0184**	1.1498	1.0335
ductal/1804 cells	1.1101	**1.0942**	1.2608	1.1415
endothelial/289 cells	1.4234	1.1993	**1.0913**	1.1579
delta/966 cells	1.1497	**1.0796**	1.8323	1.1575
acinar/1368 cells	1.1316	**1.0678**	1.2599	1.0900
beta/3826 cells	1.0359	**1.0248**	1.1049	1.0413
gamma/656 cells	1.0981	**1.0492**	1.8292	1.1698
mesenchymal/107 cells	1.7454	**1.4607**	1.8831	1.8105
epsilon/28 cells	1.8515	2.1167	1.9766	**1.8270**
MHC/5 cells	2.1894	**1.7233**	2.7904	2.1383
stellate/511 cells	**1.3191**	1.3211	1.5481	1.3785
mast/32 cells	1.5550	2.0847	**1.2647**	1.3548
macrophage/55 cells	1.9468	2.0541	**1.6261**	1.9124
schwann/13 cells	3.0230	**2.4232**	3.5840	2.8155
tcell/7 cells	3.1079	3.6881	3.0163	**2.8862**
Overall cLISI	1.0981	**1.0675**	1.2619	1.0964

Table S7: Cell type and overall cLISI scores of the human pancreas dataset (dataset 7) after removing batch effects using SCIBER, Harmony, LIGER and Seurat. The first column contains the cell types with the corresponding number of cells. A bold number stands for the best score in that row.

Cell type cLISI	SCIBER	Harmony	LIGER	Seurat
293t/4490 cells	1.1168	**1.0058**	1.4961	1.1623
jurkat/5041 cells	1.0526	**1.0030**	1.5146	1.4777
Overall cLISI	1.0828	**1.0043**	1.5059	1.3291

Table S8: Cell type and overall cLISI scores of the cell line dataset (dataset 8) after removing batch effects using SCIBER, Harmony, LIGER and Seurat. The first column contains the cell types with the corresponding number of cells. A bold number stands for the best score in that row.

Overall cLISI	$K = 0.8\sqrt{n_0}$	$K = \sqrt{n_0}$	$K = 1.2\sqrt{n_0}$
Dataset 1	1.1509	1.1483	1.1622
Dataset 2	1.1391	1.1398	1.1755
Dataset 3	1.1247	1.1203	1.1165
Dataset 4	1.0468	1.0503	1.0467
Dataset 5	1.0734	1.0621	1.0564
Dataset 6	1.5051	1.4901	1.4941
Dataset 7	1.0935	1.0981	1.0988
Dataset 8	1.0938	1.0828	1.1080

Table S9: Overall cLISI scores of eight datasets after removing batch effects using SCIBER with different K's, fixed $\omega = 0.5$ and $h = 75$. It can be noticed that with different K's, the cLISI scores vary little for each dataset, indicating SCIBER is robust to the choice of K.

Overall cLISI	$h = 50$	$h = 75$	$h = 100$
Dataset 1	1.1632	1.1483	1.1529
Dataset 2	1.1565	1.1398	1.1632
Dataset 3	1.1223	1.1203	1.1195
Dataset 4	1.0553	1.0503	1.0458
Dataset 5	1.0712	1.0621	1.0658
Dataset 6	1.5304	1.4901	1.4969
Dataset 7	1.0905	1.0981	1.1002
Dataset 8	1.0704	1.0828	1.0788

Table S10: Overall cLISI scores of eight datasets after removing batch effects using SCIBER with different h's, fixed $\omega = 0.5$ and $K = \sqrt{n_0}$. It can be noticed that with different h's, the cLISI scores vary little for each dataset, indicating SCIBER is robust to the choice of h.

Overall cLISI	$\omega = 0.4$	$\omega = 0.5$	$\omega = 0.6$	$\omega = 0.7$
Dataset 1	1.1590	1.1483	1.1586	1.1513
Dataset 2	1.1691	1.1398	1.1440	1.1126
Dataset 3	1.1169	1.1203	1.1203	1.1187
Dataset 4	1.0441	1.0503	1.0555	1.0572
Dataset 5	1.0548	1.0621	1.0695	1.0742
Dataset 6	1.5054	1.4901	1.5269	1.5763
Dataset 7	1.0994	1.0981	1.0956	1.0942
Dataset 8	1.0861	1.0828	1.1835	1.1981

Table S11: Overall cLISI scores of eight datasets after removing batch effects using SCIBER with different ω's, fixed $h = 75$ and $K = \sqrt{n_0}$. It can be noticed that with different ω's, the cLISI scores vary little for each dataset, indicating SCIBER is robust to the choice of ω.

6 Supplementary figures

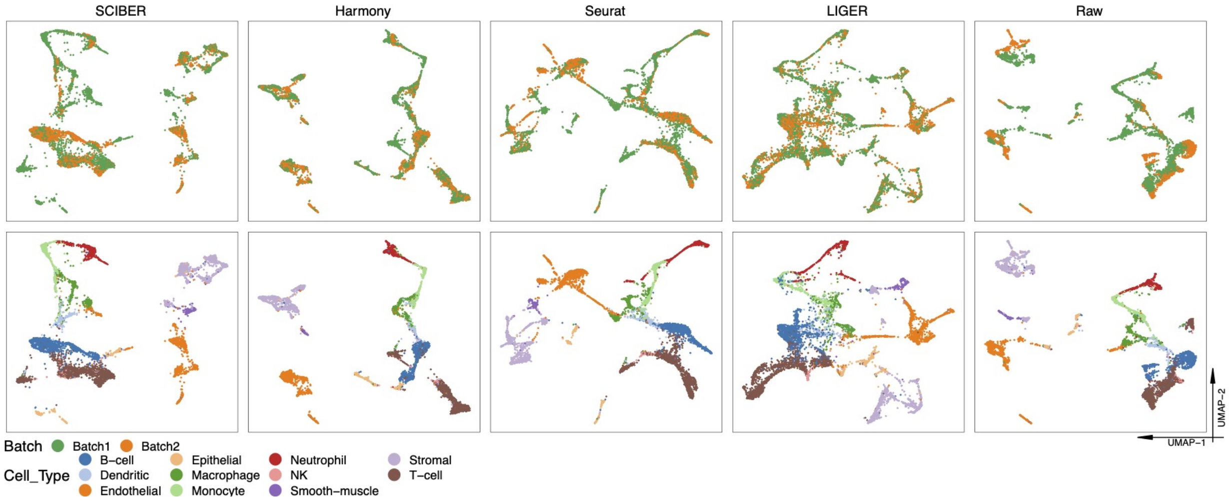

Figure S1: Scatter plots of cells from all batches of mouse cell atlas dataset (dataset 1) on a two-dimensional UMAP space. Different sub-plots show corrected data from different methods (from left to right: SCIBER, Harmony, Seurat, and LIGER), as well as the raw (uncorrected) data. Cells in the first row of sub-plots are colored according to their batches, while cells in the second row are colored according to their actual cell types.

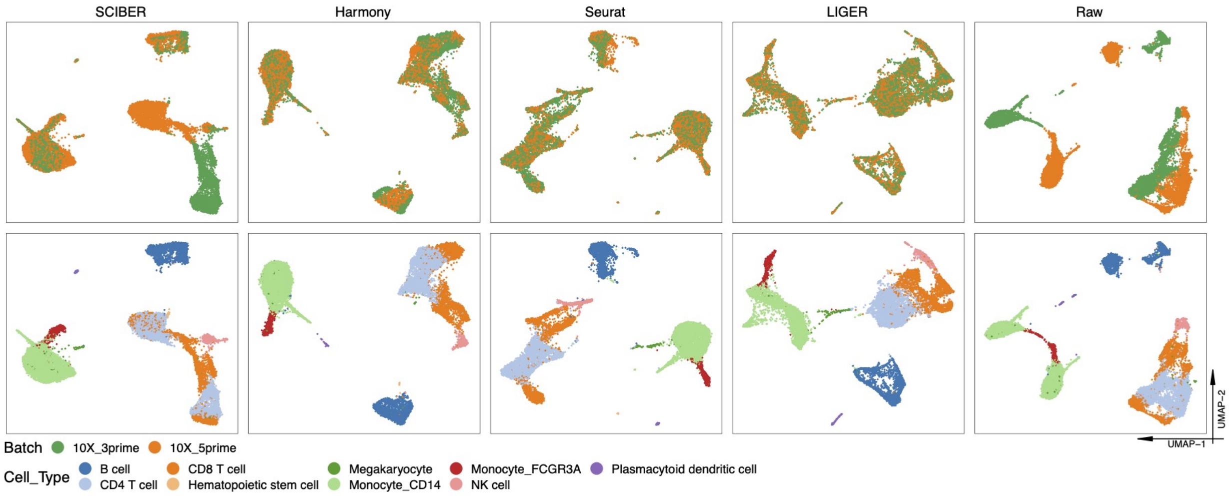

Figure S2: Scatter plots of cells from all batches of human peripheral blood mononuclear cell dataset (dataset 2) on a two-dimensional UMAP space. Different sub-plots show corrected data from different methods (from left to right: SCIBER, Harmony, Seurat, and LIGER), as well as the raw (uncorrected) data. Cells in the first row of sub-plots are colored according to their batches, while cells in the second row are colored according to their actual cell types.

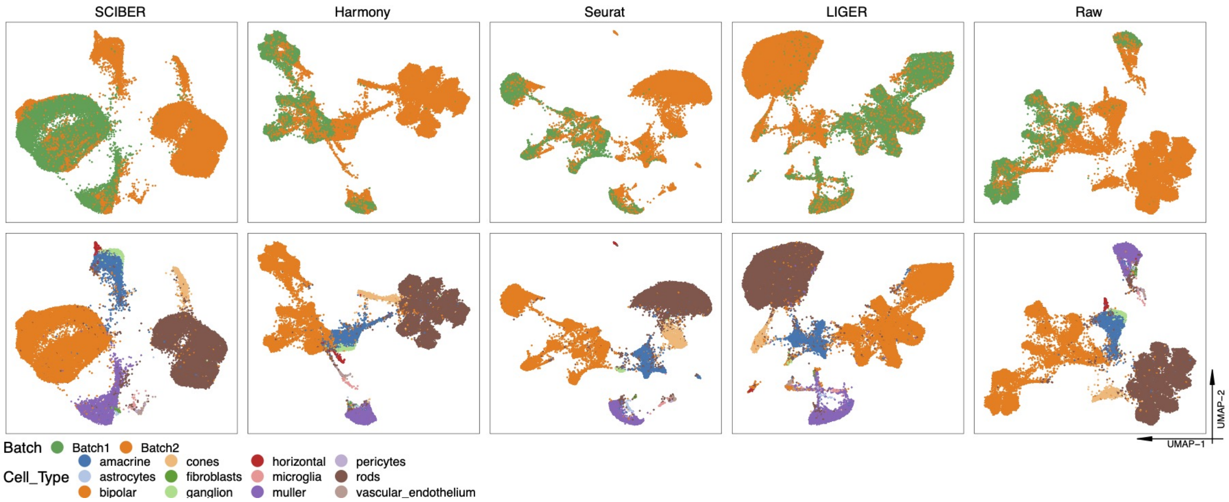

Figure S3: Scatter plots of cells from all batches of mouse retina dataset (dataset 4) on a two-dimensional UMAP space. Different sub-plots show corrected data from different methods (from left to right: SCIBER, Harmony, Seurat, and LIGER), as well as the raw (uncorrected) data. Cells in the first row of sub-plots are colored according to their batches, while cells in the second row are colored according to their actual cell types.

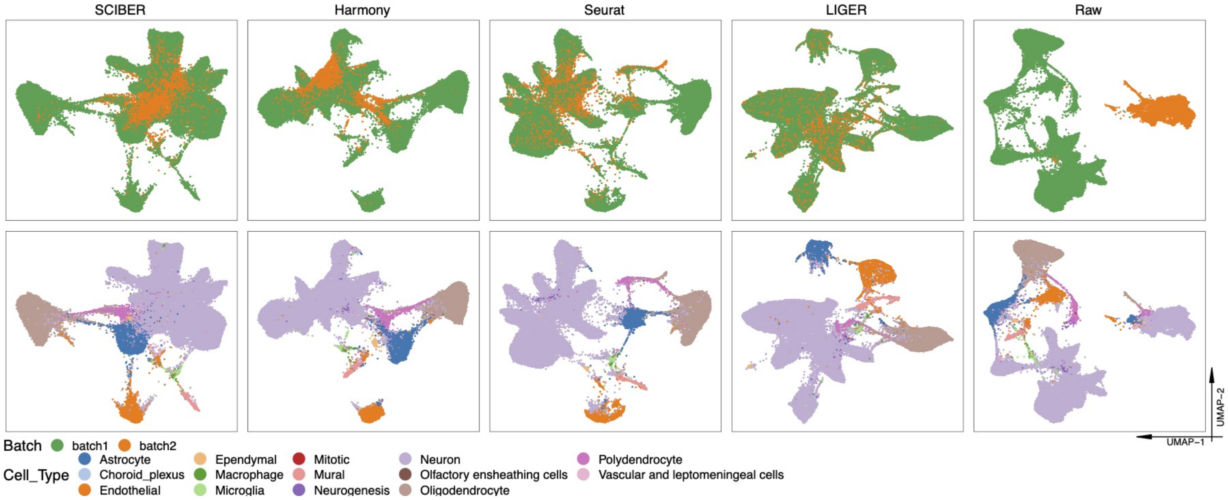

Figure S4: Scatter plots of cells from all batches of mouse brain dataset (dataset 5) on a two-dimensional UMAP space. Different sub-plots show corrected data from different methods (from left to right: SCIBER, Harmony, Seurat, and LIGER), as well as the raw (uncorrected) data. Cells in the first row of sub-plots are colored according to their batches, while cells in the second row are colored according to their actual cell types.

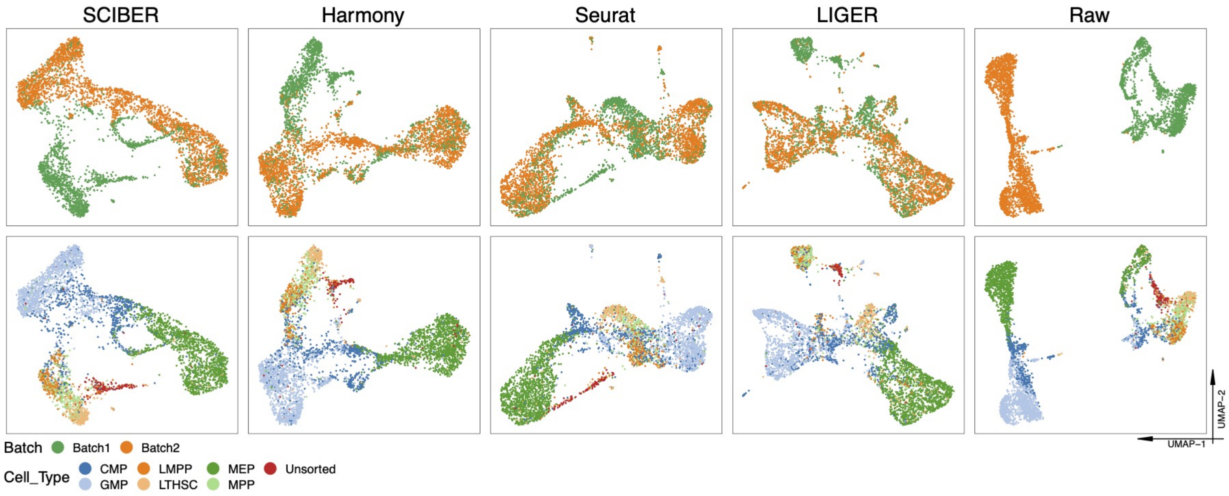

Figure S5: Scatter plots of cells from all batches of mouse haematopoietic stem and progenitor cell dataset (dataset 6) on a two-dimensional UMAP space. Different sub-plots show corrected data from different methods (from left to right: SCIBER, Harmony, Seurat, and LIGER), as well as the raw (uncorrected) data. Cells in the first row of sub-plots are colored according to their batches, while cells in the second row are colored according to their actual cell types.

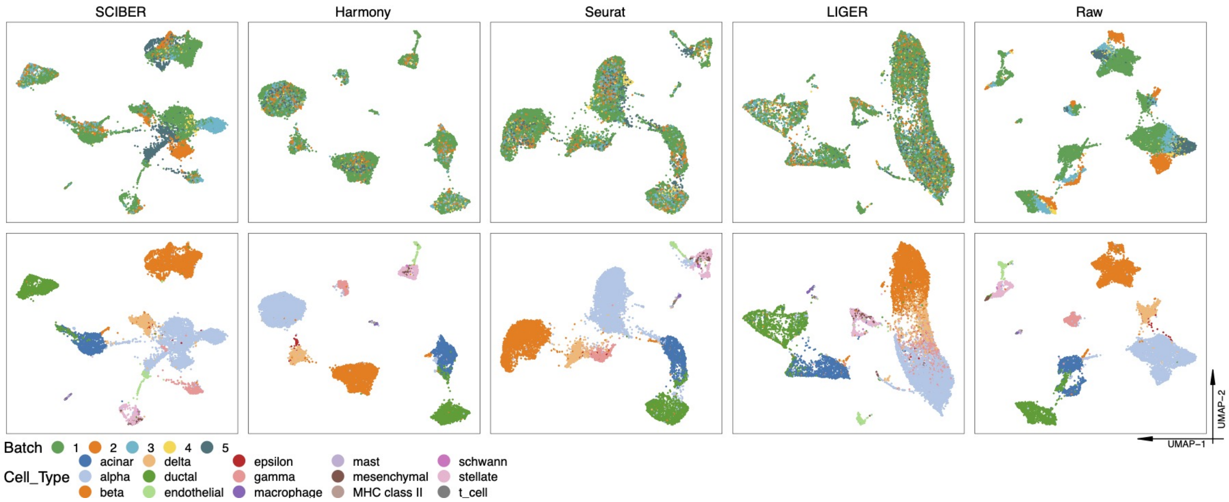

Figure S6: Scatter plots of cells from all batches of human pancreas dataset (dataset 7) on a two-dimensional UMAP space. Different sub-plots show corrected data from different methods (from left to right: SCIBER, Harmony, Seurat, and LIGER), as well as the raw (uncorrected) data. Cells in the first row of sub-plots are colored according to their batches, while cells in the second row are colored according to their actual cell types.

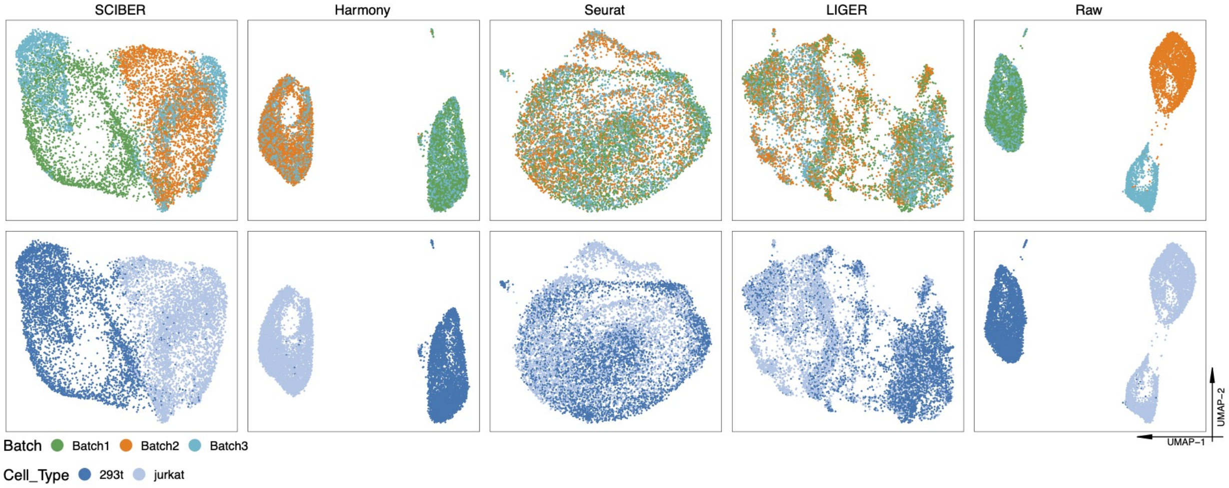

Figure S7: Scatter plots of cells from all batches of cell line dataset (dataset 8) on a two-dimensional UMAP space. Different sub-plots show corrected data from different methods (from left to right: SCIBER, Harmony, Seurat, and LIGER), as well as the raw (uncorrected) data. Cells in the first row of sub-plots are colored according to their batches, while cells in the second row are colored according to their actual cell types.

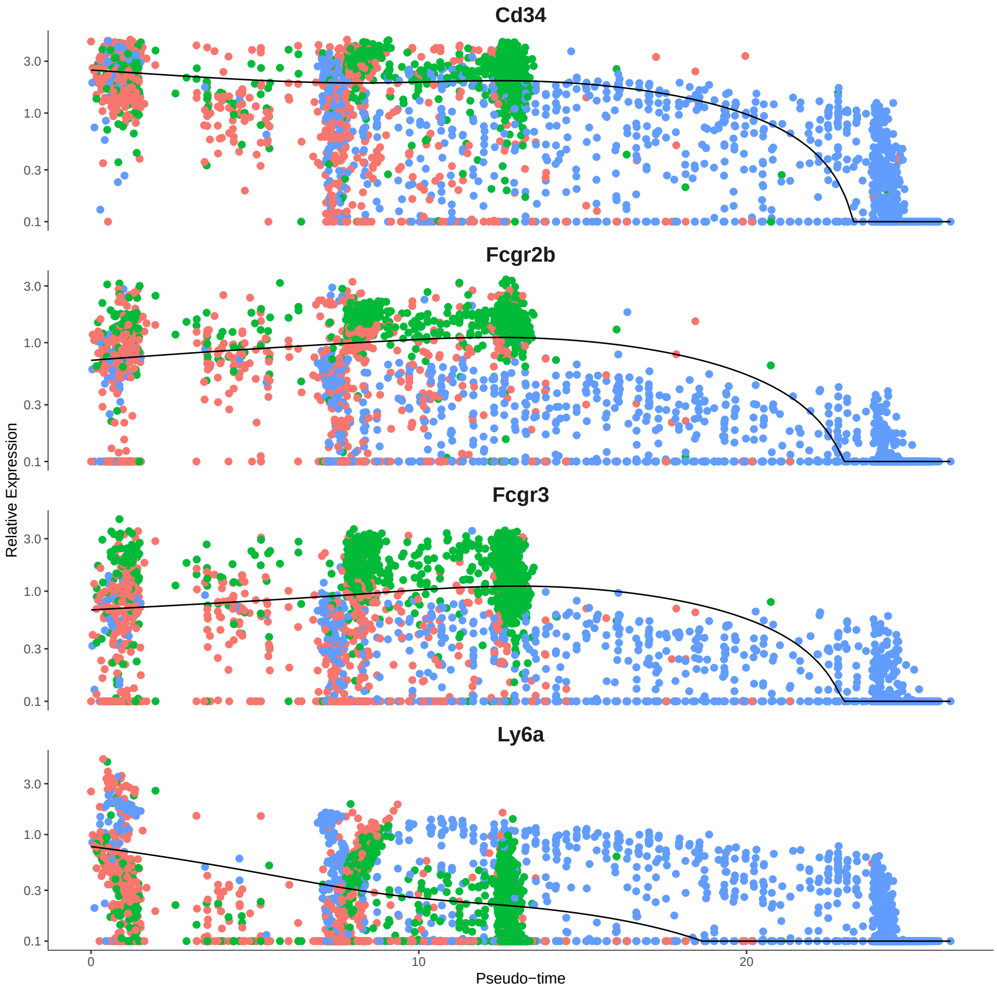

Figure S8: Gene expression level of marker genes shared by CMP, GMP and MEP based on the output data of SCIBER. The points are colored according to their actual cell types and consistent with the colors in Fig. 4. The black curve is the fitted expression level along the pseudotime.

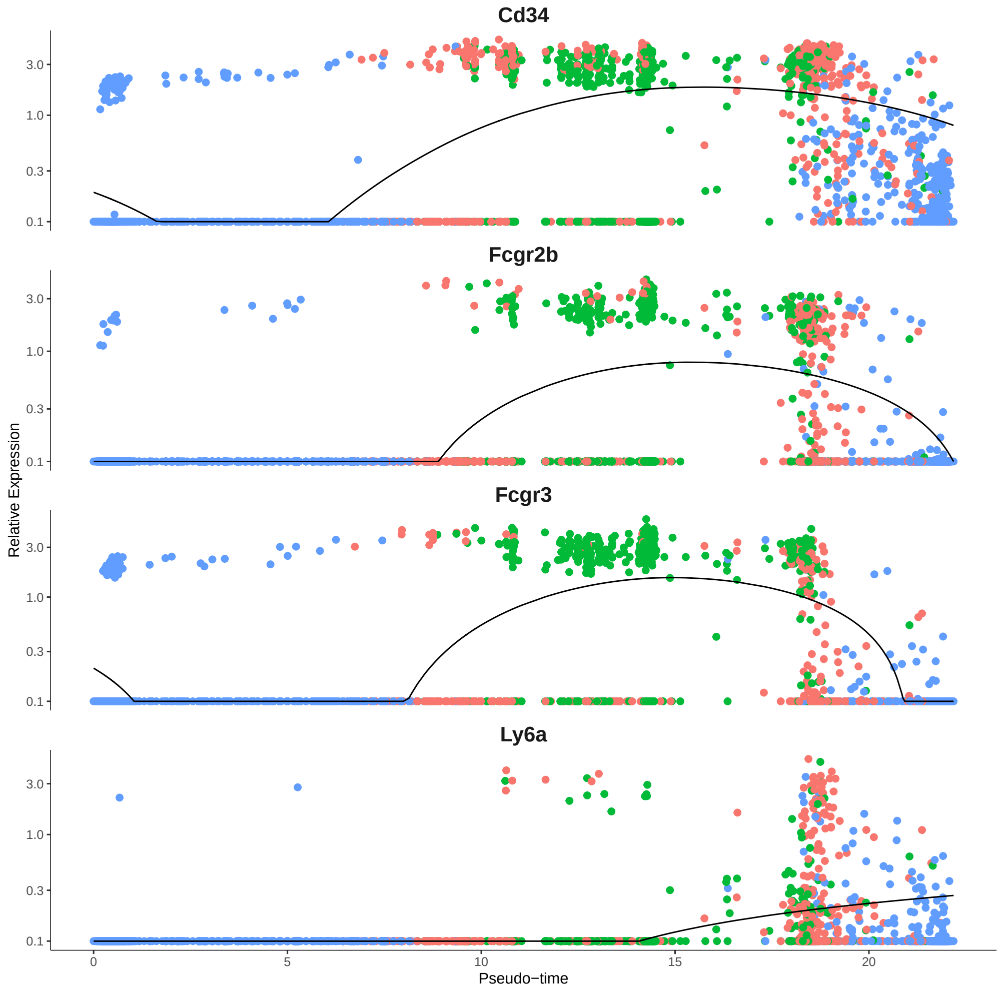

Figure S9: Gene expression level of marker genes shared by CMP, GMP and MEP based on the raw data. The points are colored according to their actual cell types and consistent with the colors in Fig. 4. The black curve is the fitted expression level along the pseudotime.

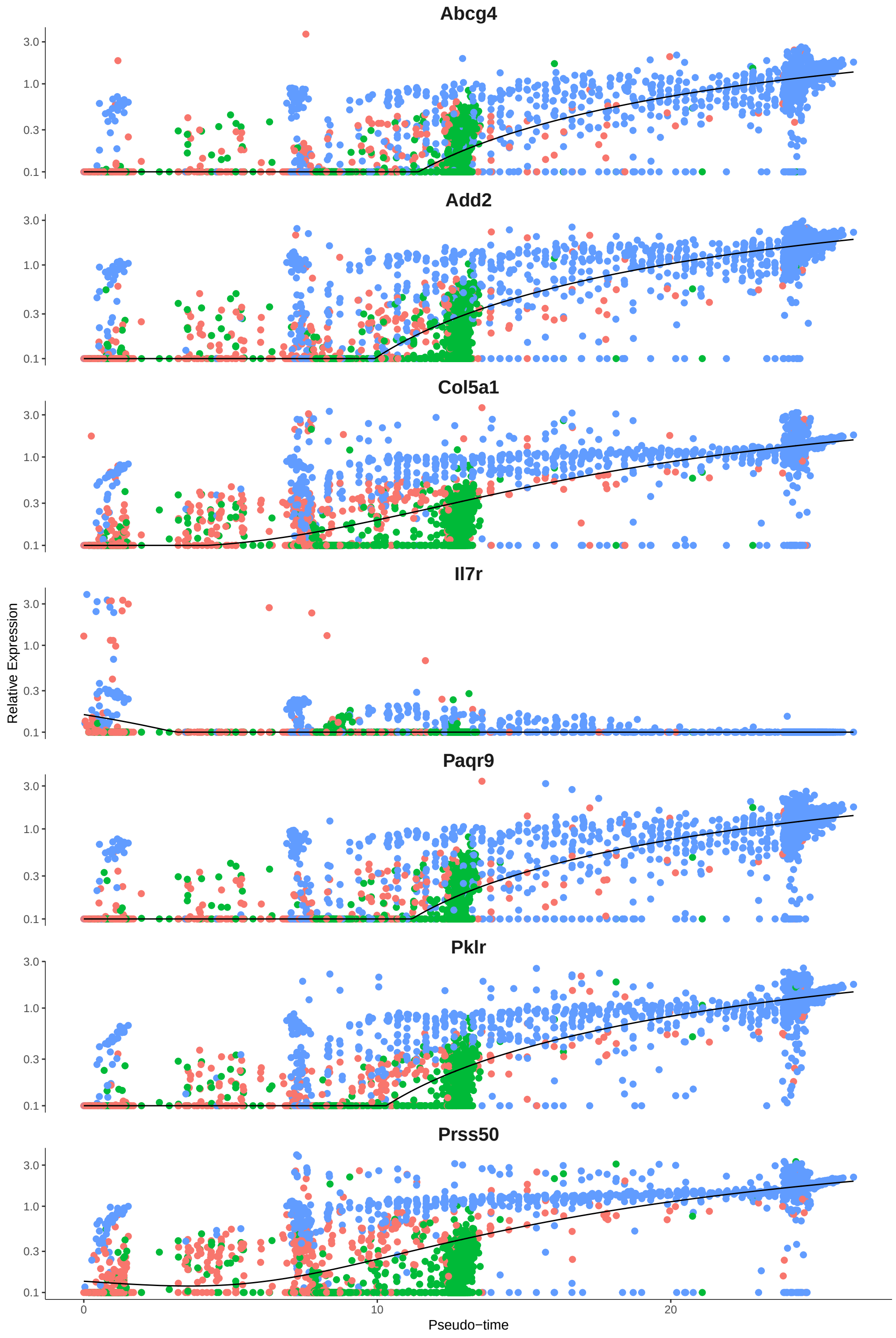

Figure S10: Gene expression level of marker genes for MEP only based on the output data of SCIBER. The points are colored according to their actual cell types and consistent with the colors in Fig. 4. The black curve is the fitted expression level along the pseudotime.

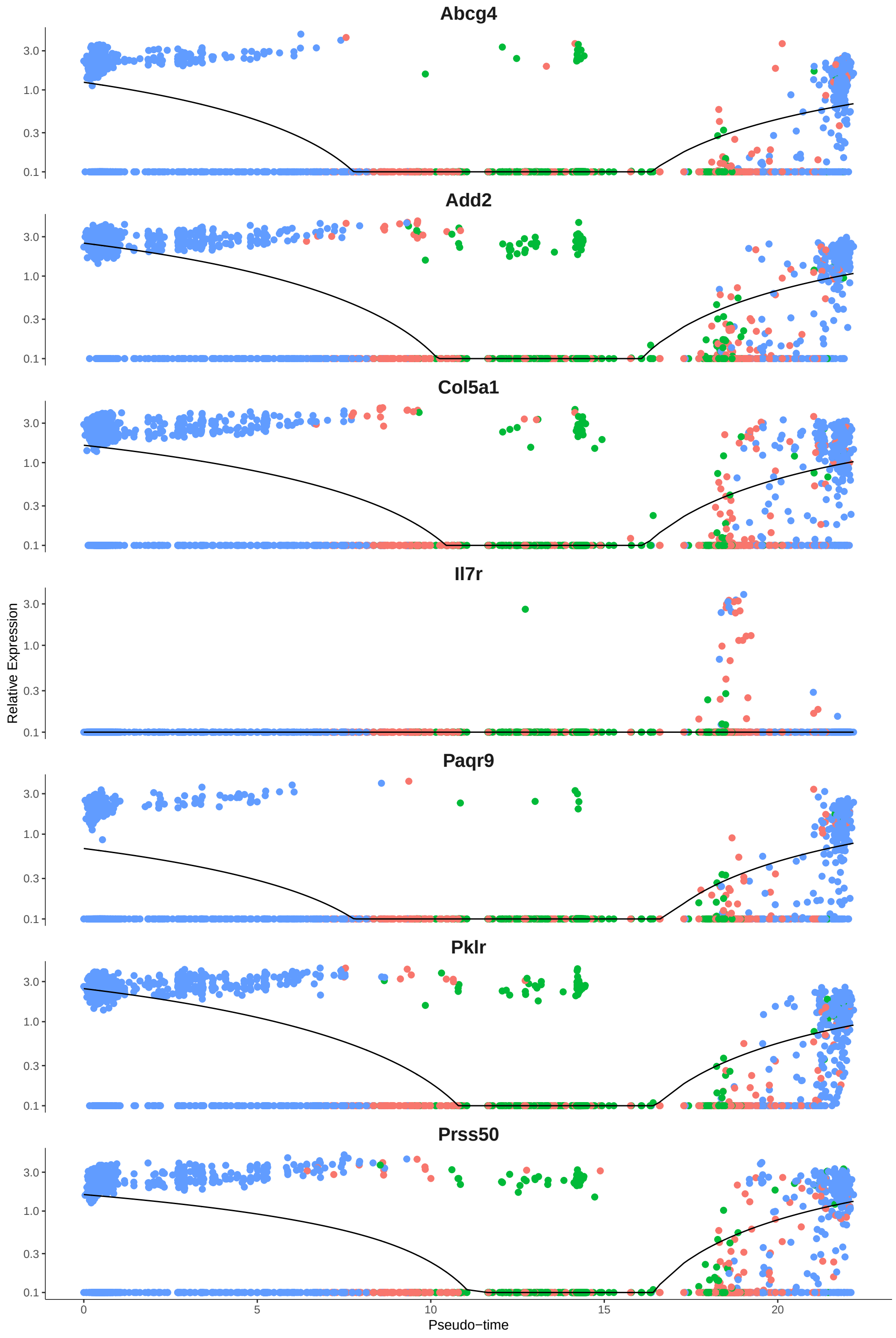

Figure S11: Gene expression level of marker genes for MEP only based on the raw data. The points are colored according to their actual cell types and consistent with the colors in Fig. 4. The black curve is the fitted expression level along the pseudotime.

References

[1] Maayan Baron, Adrian Veres, Samuel L Wolock, Aubrey L Faust, Renaud Gaujoux, Amedeo Vetere, Jennifer Hyoje Ryu, Bridget K Wagner, Shai S Shen-Orr, Allon M Klein, et al. A single-cell transcriptomic map of the human and mouse pancreas reveals inter-and intra-cell population structure. *Cell systems*, 3(4):346–360, 2016.

[2] Tabula Muris Consortium et al. Single-cell transcriptomics of 20 mouse organs creates a tabula muris. *Nature*, 562(7727):367, 2018.

[3] Laleh Haghverdi, Aaron TL Lun, Michael D Morgan, and John C Marioni. Batch effects in single-cell rna-sequencing data are corrected by matching mutual nearest neighbors. *Nature biotechnology*, 36(5):421–427, 2018.

[4] Xiaoping Han, Renying Wang, Yincong Zhou, Lijiang Fei, Huiyu Sun, Shujing Lai, Assieh Saadatpour, Ziming Zhou, Haide Chen, Fang Ye, et al. Mapping the mouse cell atlas by microwell-seq. *Cell*, 172(5):1091–1107, 2018.

[5] Brian Hie, Bryan Bryson, and Bonnie Berger. Efficient integration of heterogeneous single-cell transcriptomes using scanorama. *Nature biotechnology*, 37(6):685–691, 2019.

[6] Lawrence Hubert and Phipps Arabie. Comparing partitions. *Journal of classification*, 2(1):193–218, 1985.

[7] Ilya Korsunsky, Nghia Millard, Jean Fan, Kamil Slowikowski, Fan Zhang, Kevin Wei, Yuriy Baglaenko, Michael Brenner, Po-ru Loh, and Soumya Raychaudhuri. Fast, sensitive and accurate integration of single-cell data with harmony. *Nature methods*, pages 1–8, 2019.

[8] Evan Z Macosko, Anindita Basu, Rahul Satija, James Nemesh, Karthik Shekhar, Melissa Goldman, Itay Tirosh, Allison R Bialas, Nolan Kamitaki, Emily M Marter-

steck, et al. Highly parallel genome-wide expression profiling of individual cells using nanoliter droplets. *Cell*, 161(5):1202–1214, 2015.

[9] Mauro J Muraro, Gitanjali Dharmadhikari, Dominic Grün, Nathalie Groen, Tim Dielen, Erik Jansen, Leon van Gurp, Marten A Engelse, Francoise Carlotti, Eelco JP de Koning, et al. A single-cell transcriptome atlas of the human pancreas. *Cell systems*, 3(4):385–394, 2016.

[10] Sonia Nestorowa, Fiona K Hamey, Blanca Pijuan Sala, Evangelia Diamanti, Mairi Shepherd, Elisa Laurenti, Nicola K Wilson, David G Kent, and Berthold Göttgens. A single-cell resolution map of mouse hematopoietic stem and progenitor cell differentiation. *Blood, The Journal of the American Society of Hematology*, 128(8):e20–e31, 2016.

[11] Franziska Paul, Ya'ara Arkin, Amir Giladi, Diego Adhemar Jaitin, Ephraim Kenigsberg, Hadas Keren-Shaul, Deborah Winter, David Lara-Astiaso, Meital Gury, Assaf Weiner, et al. Transcriptional heterogeneity and lineage commitment in myeloid progenitors. *Cell*, 163(7):1663–1677, 2015.

[12] Krzysztof Polański, Matthew D Young, Zhichao Miao, Kerstin B Meyer, Sarah A Teichmann, and Jong-Eun Park. Bbknn: fast batch alignment of single cell transcriptomes. *Bioinformatics*, 36(3):964–965, 2020.

[13] Alexander B Rosenberg, Charles M Roco, Richard A Muscat, Anna Kuchina, Paul Sample, Zizhen Yao, Lucas T Graybuck, David J Peeler, Sumit Mukherjee, Wei Chen, et al. Single-cell profiling of the developing mouse brain and spinal cord with split-pool barcoding. *Science*, 360(6385):176–182, 2018.

[14] Peter J Rousseeuw. Silhouettes: a graphical aid to the interpretation and validation of cluster analysis. *Journal of computational and applied mathematics*, 20:53–65, 1987.

[15] Arpiar Saunders, Evan Z Macosko, Alec Wysoker, Melissa Goldman, Fenna M Krienen, Heather de Rivera, Elizabeth Bien, Matthew Baum, Laura Bortolin, Shuyu Wang, et al. Molecular diversity and specializations among the cells of the adult mouse brain. *Cell*, 174(4):1015–1030, 2018.

[16] Åsa Segerstolpe, Athanasia Palasantza, Pernilla Eliasson, Eva-Marie Andersson, Anne-Christine Andréasson, Xiaoyan Sun, Simone Picelli, Alan Sabirsh, Maryam Clausen, Magnus K Bjursell, et al. Single-cell transcriptome profiling of human pancreatic islets in health and type 2 diabetes. *Cell metabolism*, 24(4):593–607, 2016.

[17] Karthik Shekhar, Sylvain W Lapan, Irene E Whitney, Nicholas M Tran, Evan Z Macosko, Monika Kowalczyk, Xian Adiconis, Joshua Z Levin, James Nemesh, Melissa Goldman, et al. Comprehensive classification of retinal bipolar neurons by single-cell transcriptomics. *Cell*, 166(5):1308–1323, 2016.

[18] Tim Stuart, Andrew Butler, Paul Hoffman, Christoph Hafemeister, Efthymia Papalexi, William M Mauck III, Yuhan Hao, Marlon Stoeckius, Peter Smibert, and Rahul Satija. Comprehensive integration of single-cell data. *Cell*, 177(7):1888–1902, 2019.

[19] Hoa Thi Nhu Tran, Kok Siong Ang, Marion Chevrier, Xiaomeng Zhang, Nicole Yee Shin Lee, Michelle Goh, and Jinmiao Chen. A benchmark of batch-effect correction methods for single-cell rna sequencing data. *Genome biology*, 21(1):1–32, 2020.

[20] Cole Trapnell, Davide Cacchiarelli, Jonna Grimsby, Prapti Pokharel, Shuqiang Li, Michael Morse, Niall J Lennon, Kenneth J Livak, Tarjei S Mikkelsen, and John L Rinn. The dynamics and regulators of cell fate decisions are revealed by pseudotemporal ordering of single cells. *Nature biotechnology*, 32(4):381–386, 2014.

[21] Alexandra-Chloé Villani, Rahul Satija, Gary Reynolds, Siranush Sarkizova, Karthik Shekhar, James Fletcher, Morgane Griesbeck, Andrew Butler, Shiwei Zheng, Suzan

Lazo, et al. Single-cell rna-seq reveals new types of human blood dendritic cells, monocytes, and progenitors. *Science*, 356(6335), 2017.

[22] Yue J Wang, Jonathan Schug, Kyoung-Jae Won, Chengyang Liu, Ali Naji, Dana Avrahami, Maria L Golson, and Klaus H Kaestner. Single-cell transcriptomics of the human endocrine pancreas. *Diabetes*, 65(10):3028–3038, 2016.

[23] Joshua Welch, Velina Kozareva, Ashley Ferreira, Charles Vanderburg, Carly Martin, and Evan Macosko. Integrative inference of brain cell similarities and differences from single-cell genomics. *bioRxiv*, page 459891, 2018.

[24] Yurong Xin, Jinrang Kim, Haruka Okamoto, Min Ni, Yi Wei, Christina Adler, Andrew J Murphy, George D Yancopoulos, Calvin Lin, and Jesper Gromada. Rna sequencing of single human islet cells reveals type 2 diabetes genes. *Cell metabolism*, 24(4):608–615, 2016.

[25] Grace XY Zheng, Jessica M Terry, Phillip Belgrader, Paul Ryvkin, Zachary W Bent, Ryan Wilson, Solongo B Ziraldo, Tobias D Wheeler, Geoff P McDermott, Junjie Zhu, et al. Massively parallel digital transcriptional profiling of single cells. *Nature communications*, 8(1):1–12, 2017.
